# Chloroplast DNA insights into the phylogenetic position and anagenetic speciation of *Phedimus takesimensis* (Crassulaceae) on Ulleung and Dokdo Islands, Korea

**DOI:** 10.1371/journal.pone.0239734

**Published:** 2020-09-28

**Authors:** Hee-Seung Seo, Seon-Hee Kim, Seung-Chul Kim

**Affiliations:** Department of Biological Sciences, Sungkyunkwan University, Suwon, Gyeonggi-do, Korea; Kunming Institute of Botany, Chinese Academy of Sciences, CHINA

## Abstract

*Phedimus takesimensis* (Ulleungdo flat-leaved stonecrop) is endemic to Ulleung and Dokdo Islands off the east coast of the Korean Peninsula. It was suggested that *P*. *takesimensis* originated via anagenetic speciation from the continental progenitor species *P*. *kamtchaticus* or *P*. *aizoon*. However, little is known of the phylogenetic relationships and population genetic structure among species of *Phedimus* in the Korean Peninsula and Ulleung/Dokdo Islands. We inferred the phylogenetic relationships among congeneric species in Korea based on nuclear ribosomal DNA internal transcribed spacer and chloroplast noncoding regions. We also sampled extensively for *P*. *takesimensis* on Ulleung Island and the continental species, *P*. *kamtschaticus* and *P*. *aizoon*, to assess the genetic consequences of anagenetic speciation. We found (1) the monophyly of *P*. *takesimensis*, (2) no apparent reduction in genetic diversity in anagenetically derived *P*. *takesimensis* compared to the continental progenitor species, (3) apparent population genetic structuring of *P*. *takesimensis*, and (4) two separate colonization events for the origin of the Dokdo Island population. This study contributes to our understanding of the genetic consequences of anagenetic speciation on Ulleung Island.

## Introduction

The unique and diverse flora and fauna of the oceanic archipelagos are excellent model systems to investigate patterns and processes of evolution; thus, oceanic islands are often considered as natural laboratories [1–5]. In particular, numerous insular endemic plant lineages with spectacular morphological, anatomical, and ecological diversity in the Pacific and Atlantic Oceans provide ample opportunities for naturalists to uncover their fascinating natural histories on islands (e.g., the silversword alliance of the Hawaiian Islands [6,7]; *Echium* of the Canary Islands [8]; *Dendroseris* and *Robinsonia* of the Juan Fernandez Islands [9–11]; the woody *Sonchus* alliance of the Canary Islands [12]). Considering the fact that approximately 25% of the extant vascular plant species occur on islands, of which a large portion are highly vulnerable to natural and anthropogenic threats, it is critical and urgent for plant systematists to discover hidden diversity and generate base line information for the conservation of critical endemic lineages [13–15]. Detailed systematic and ecological studies combined with the use of high throughput genomic sequence data and a diverse array of analytical tools are essential to elucidate the complex evolutionary history and facilitate the conservation of numerous endemic plant lineages that are shaped by different evolutionary processes (e.g., cladogenesis, anagenesis, and reticulation) [16].

Ulleung Island, an oceanic volcanic island in the East Sea, is located approximately 137 km east of the Korean Peninsula and has never been connected to the adjacent continental land mass. It is estimated to be approximately 1.8 million years old (myr) with a total area of 73 km^2^ and a highest peak of 984 m above sea level [17]. Albeit small, Ulleung Island is home to approximately 500 native vascular plant species, approximately 36 of which are endemic species primarily driven via anagenetic speciation. During anagenetic speciation, an initial founder lineage simply transforms genetically and morphologically through time without further specific differentiation [18,19]. The diversity estimates based on the endemic plant species per 100 km^2^ and the number of plant species per km^2^ are much higher than those of the Hawaiian and Canary Islands [20,21]. Given the important role of anagenetic speciation (also known as phyletic or gradual speciation; see also anacladogenesis, [22]) for explaining the origin of the majority of plant endemics, Ulleung Island has been recognized as an ideal place to investigate this particular mode of speciation among other oceanic archipelagos.

Of the nearly 40 currently recognized endemic plant lineages on Ulleung Island that presumably have their origins in anagenetic speciation, *Phedimus takesimensis* (Nakai) 't Hart (= *Sedum takesimense* Nakai) represents an endemic species in the family Crassulaceae of the core eudicots clade. The genus *Phedimus*, with around 20 species primarily in Asia and Europe, represents one lineage of non-monophyletic subfamily Sedoideae [23]. Traditionally, species of *Phedimus* have been treated as members of *Sedum*, but a recent phylogenetic study strongly supported the monophyly of *Phedimus* and its segregation from the genus *Sedum* [24]. *Phedimus takesimensis* occurs exclusively on the seaside and sunny slopes of Ulleung and Dokdo Islands in Korea. It is a robust perennial herb with overwintering shoots on the lower part of stem, erect or decumbent with a height of 20‒50 cm [21]. *Phedimus takesimensis* is morphologically similar to *P*. *aizoon* (L.) ‘t Hart, but the former species can be distinguished from the latter primarily based on overwintering shoots on the lower part of the stems and much shorter internodes, giving the appearance of tightly clustered leaves ([25]; S-C. Kim, personal observation). In addition, *P*. *aizoon* does not branch much and has yellow anthers, while *P*. *takesimensis* is much more branched, like *P*. *kamtschaticus*, with reddish yellow anthers [26]. *Phedimus kamtschaticus*, which occurs commonly in northeastern Asia, is morphologically similar to *P*. *aizoon*, with intermediate morphological forms between the two species, but it can be distinguished based on the presence of more branched stems. Given the morphological similarities among these taxa and significant variation within each taxon, there is substantial confusion and numerous taxonomic classifications abound. For example, in addition to the newly described species *Sedum zokuriense* Nakai [= *Phedimus zokuriensis* (Nakai) ‘t Hart] from Mt. Seokrisan [27], *S*. *takesimense* was recognized as infraspecific taxon of *S*. *kamtschaticum*, i.e., *S*. *kamtschaticum* var. *takesimense* and *S*. *kamtschaticum* var. *zokuriensis* [28]. However, a distinct species status was proposed based on floristic and taxonomic studies [26,29]. Several infraspecific taxa (up to seven varieties) within *S*. *aizoon* were recognized (see the various treatments described in [26]), while Lee [30] recognized only one taxon. In addition, a morphometric study of the subgenus *Aizoon* in Korea suggested that although they formed distinct clusters within *S*. *kamtschaticum* in a broad sense, the three species *S*. *takesimense*, *S*. *zokuriense*, and *S*. *ellacombianum* [= *Phedimus ellacombianus* (Praeger) ‘t Hart] could be recognized as infraspecific taxa of *S*. *kamtschaticum* [31]. Interspecific gene flows and hybridizations among the species of the subgenus *Aizoon* in Korea (i.e., *S*. *latiovalifolium*, *S*. *kamtschaticum*, and *S*. *aizoon*) were also documented [32]. Furthermore, extensive polyploidy and aneuploidy in members of the subgenus *Aizoon* were demonstrated, partly explaining a certain degree of morphological variation [33–35].

Considering the origin of endemic *Phedimus takesimensis* on Ulleung and Dokdo Islands in the East Sea, few continental species could be proposed as likely candidate progenitor species. Based on a morphological analysis, the two species *S*. *kamtschaticum* [= *P*. *kamtschaticus*] and *S*. *ellacombianum* [= *P*. *ellacombianus*] were clustered with *S*. *takesimense*, while *S*. *aizoon* [= *P*. *aizoon*] formed a distinct and separate cluster from the *S*. *kamtschaticum* group [31]. While *P*. *kamtschaticus* occurs widely in the Korean Peninsula and neighboring regions (eastern China, Russia, and Japan), *P*. *ellacombianus* is restricted to the southern part of the Korean Peninsula [26]. It was shown, based on very limited sampling, that *P*. *aizoon* is closely related to *P*. *takesimensis* genetically based on RAPDs (randomly amplified polymorphic DNAs) [36]. The first assessment of the genetic diversity of *P*. *takesimensis* and its relationship to a continental progenitor was based on one noncoding chloroplast (cp) region (*trn*L-F) [37]. Based on a total of 32 individuals (30 individuals of *P*. *takesimensis* and two individuals of *P*. *kamtschaticus*), two chlorotypes were found; “type01” was found exclusively on Ulleung Island (15 individuals), while “type02” was found in 17 individuals from the Ulleung and Dokdo Islands. In addition, two individuals of *P*. *kamtschaticus*—as a continental progenitor—were similar to the chlorotype “type02” in that they had a 6-bp deletion but were distinguished by a single point mutation [37]. Recently, a *Phedimus* cultivar identification study, including several purported wild progenitor species, demonstrated unresolved species relationships within the genus [38]. For example, it was shown that the highly polymorphic *P*. *takesimensis* was unresolved with *P*. *middendorffianus* and *P*. *kamtschaticus* based on nuclear ribosomal DNA (nrDNA) internal transcribed spacer (ITS) sequences, while one cp noncoding region (*psb*A-*trn*H) sequence suggested an unresolved relationship with *P*. *aizoon*, *P*. *kamtschaticus*, and *P*. *middendorffianus*. It seems likely that this lack of clear species relationships could be attributed to various factors, such as polyploidization, aneuploidization, interspecific gene flow, concerted evolution of nrDNA, incomplete lineage sorting of ancient polymorphisms, lack of sufficient cpDNA variation, and any combination of these. While precise species relationships within the genus remain to be determined, it is reasonable to consider that *P*. *kamtschaticus* is one of plausible continental progenitor species of *P*. *takesimensis* on Ulleung Island based on morphological characteristics.

In this study, we sampled extensively *P*. *takesimensis* on Ulleung and Dokdo Islands and estimated their genetic variation and population structure based on five cpDNA noncoding regions. Then, we compared the genetic diversity and population genetic structure of *P*. *takesimensis* with two representative continental progenitor species (*P*. *kamtschaticus* and *P*. *aizoon*). Lastly, we took a phylogenetic approach to determine the phylogenetic position of *P*. *takesimensis* among the congeneric species occurring in Korea. Based on extensive sampling and the combination of population genetics and phylogenetic approaches, we hope to (1) estimate the level of genetic variation between insular endemic and purported continental progenitor species, (2) reveal the population genetic structure of *P*. *takesimensis* on Ulleung and Dokdo Islands, (3) determine the relationship between Ulleung and Dokdo Island populations of *P*. *takesimensis*, and (4) infer the relationship between insular derived endemic *P*. *takesimensis* and continental congeneric species.

## Materials and methods

### Plant materials

**Fig 1:**
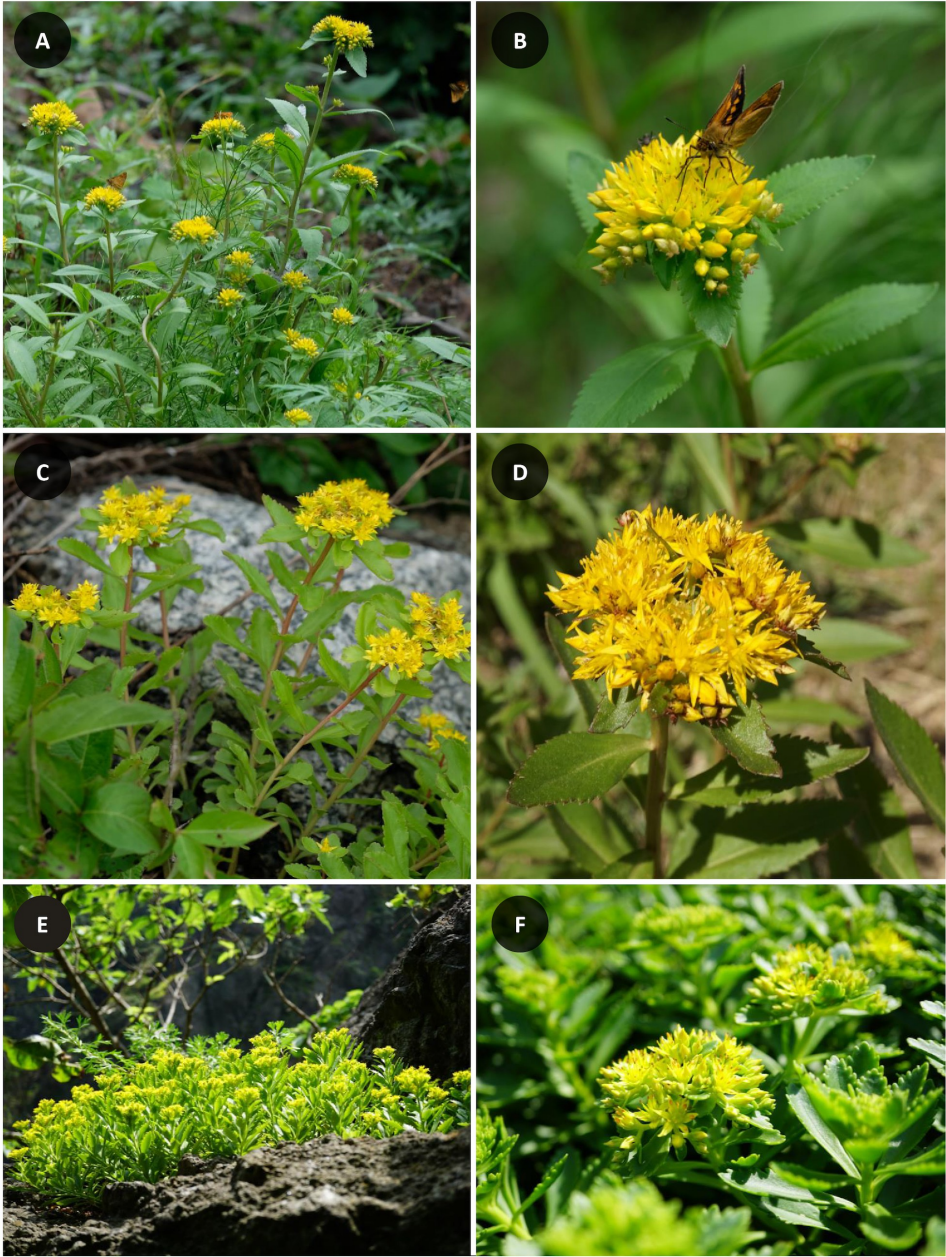
Photographs of three *Phedimus* species in Korea. (a) and (b) *P*. *aizoon* (Yang-gu, Gangwon-do); (c) and (d) *P*. *kamtschaticus* (Yang-gu, Gangwon-do); (e) and (f) *P*. *takesimensis* (Ulleung Island, Gyeongsangbuk-do).

For the phylogenetic analysis of the genus *Phedimus* in Korea, we sampled nine accessions of *P*. *aizoon*, 10 accessions of *P*. *kamtschaticus*, 15 accessions of *P*. *takesimensis*, and two accessions of *P*. *ellacombianus* (Fig 1 and Table 1). In the case of *P*. *kamtschaticus*, we sampled one accession from a representative locality in the Korean Peninsula: Gangwon-do Province (three locations: Mt. Hambaek, Sokcho-si, and Cheorwon-gun), Gyeonggi-do Province (two locations: Mt. Ungil and Mt. Munsu), Chungcheongnam-do Province (one location: Taean-gun), Gyeongsanbuk-do Province (one location: Pohang-si), Jeollanam-do Province (one location: Mudeungsan National Park), and Jeollabuk-do Province (two locations: Mt. Deogyu and Mt. Jiri). For *P*. *latiovalifolius*, which is endemic to Korea, we sampled a total of 10 accessions from Gangwon-do Province (Taebaek-si) and one accession from the same 15 populations sampled for the population genetic study for *P*. *takesimensis* (Table 1). No permits were required to collect the species in Korea given their conservation status. For the outgroups, we selected *Rhodiola* and *Sedum* sensu stricto, and DNA sequences of the representative species were obtained from GenBank (Table 1). The population codes for the sampling locations of each species are given in Table 1.

**Table 1:** A list of *Phedimus* species sampled in this study and their sampling locations. Vouchers were deposited at Ha Eun Herbarium (SKK) of Sungkyunkwan University.

Population code	Locality	GPS	Altitude (m)	N/I	Voucher
***Phedimus aizoon* (three populations; 16 individuals)**					
HWA	Mt. Hwaak, Sanae-myeon, Hwacheon-gun, Gangwon-do	38°00'04.73"N 127°31'36.28"E	1,036 m	8 (4)*	SEO0085 SKK
TAE	Geum Dae Peak, Geumdaebong-gil, Gohan-eup, Jeongseon-gun, Gangwon-do	37°12'15.6"N 128°54'54.6"E	1,289 m	7 (4)*	SKK 150804903
SUN**	Sunjaryung, Pyeongchang-gun, Gangwon-do	37°41'23.3"N 128°45'28.1"E	1,002 m	1	SEO0072 SKK
***Phedimus ellacombeanus* (one population; two individuals)**					
SO**	Sochi island, Namhae-gun, Gyeongsangnam-do	34°40'39.2"N 127°56'55.8"E	-	2	20170614001
***Phedimus kamtschaticus* (10 populations; 79 individuals)**					
AN	Angalume beach, Taean-gun, Chungcheongnam-do	36°42'19.2"N 126°09'22.8"E	5 m	8	SEO0061 SKK
DUK	Mt. Deogyu, Muju-gun, Jeollabuk-do	35°52'20.3"N 127°44'37.8"E	1,253 m	8	SEO0058 SKK
GOO	Guryongpo Beach, Pohang-si, Gyeongsangbuk-do	36°01'33.76"N 129°35'10.42"E	18 m	8	SEO0068 SKK
HAM	Mt. Hambaek, Jeongseon-gun, Gangwon-do	37°09'16.6"N 128°54'53.4"E	1,334 m	8	SEO0055 SKK
JI	Mt. Jiri, Namwon-si, Jeollabuk-do	35°17'46.6"N 127°31'46.9"E	1,428 m	8	SEO0059 SKK
MUD	Mudeungsan National Park Gwangju-si, Jeollanam-do	35°07'07.6"N 127°59'23.9"E	75 m	8	SEO0051 SKK
MUN	Mt. Munsu, Gimpo-si, Gyeonggi-do	37°44'21.4"N 126°32'54.7"E	344 m	8	SEO0063 SKK
SAM	Sambuyeon waterfall, Cheorwon-gun, Gangwon-do	38°08'20.2"N 127°20'05.2"E	232 m	7	SEO0069 SKK
UN	Mt. Ungil, Namyangju-si, Gyeonggi-do	37°34'15.6"N 127°18'07.5"E	381 m	8	SEO0053 SKK
YON	Yeonggeumjeong, Sokcho-si, Gangwon-do	38°12'46.95"N 128°36'11.86"E	0 m	8	SEO0065 SKK
***Phedimus latiovalifolius* (one population; 10 individuals)**					
GUE*	Geum Dae Peak, Taebaek-si, Gangwon-do	37°12'30.0"N 128°54'56.4"E	1,337 m	10	SKK150804930
***Phedimus takesimensis* (15 populations; 148 individuals)**					
NAE	Naesujeon, Jeodong-ri, Ulleung-eup, Ulleung-gun, Gyeongsangbuk-do	37°30'27.3"N 130°54'33.9"E	129 m	10	SEO0001 SKK
BON	BongraeFall, Jeodong-ri, Ulleung-eup, Ulleung-gun, Gyeongsangbuk-do	37°29'52.6"N 130°53'18.2"E	296 m	10	SEO0042 SKK
CHE	Cheonbu, Cheonbu-ri, Buk-myeon, Ulleung-eup, Ulleung-gun, Gyeongsangbuk-do	37°32'20.8"N 130°52'11.3"E	18 m	10	SEO0024 SKK
CHU	Chusan, Buk-myeon, Ulleung-eup, Ulleung-gun, Gyeongsangbuk-do	37°32'02.9"N 130°51'07.0"E	260 m	10	SEO0039 SKK
DOD	Dodong, Dodong-ri, Ulleung-eup, Ulleung-gun, Gyeongsangbuk-do	37°29'05.3"N 130°54'34.9"E	99 m	10	SEO0046 SKK
DON	Dokdo Island (East Island), Dokdo-ri, Ulleung-eup, Ulleung-gun, Gyeongsangbuk-do	37°14'20.9"N 131°52'10.3"E	23 m	10	SEO0049 SKK
GUA	Guam, Namseo-ri, Seo-myeon, Ulleung-eup, Ulleung-gun, Gyeongsangbuk-do	37°28'43.1"N 130°48'32.9"E	17 m	10	SEO0031 SKK
HAK	Hakpo, Seo-myeon, Ulleung-eup, Ulleung-gun, Gyeongsangbuk-do	37°30'16.9"N 130°48'20.9"E	51 m	10	SEO0035 SKK
HYE	Hyeonpo, Hyeonpo-ri, Buk-myeon, Ulleung-eup, Ulleung-gun, Gyeongsangbuk-do	37°31'41.2"N 130°49'49.7"E	5 m	8	SEO0037 SKK
JEO	Jeodong, Jeodong-ri, Ulleung-eup, Ulleung-gun, Gyeongsangbuk-do	37°29'28.6"N 130°54'47.9"E	14 m	10	SEO0030 SKK
NAM	Namyang, Namseo-ri, Seo-myeon, Ulleung-eup, Ulleung-gun, Gyeongsangbuk-do,	37°28'01.0"N 130°50'11.6"E	12 m	10	SEO0012 SKK
SAD	Sadong, Sadong-ri, Ulleung-eup, Ulleung-gun, Gyeongsangbuk-do	37°27'31.3"N 130°52'30.6"E	28 m	10	SEO0021 SKK
SUM	Seommok, Cheonbu-ri, Buk-myeon, Ulleung-eup, Ulleung-gun, Gyeongsangbuk-do	37°32'33.5"N 130°54'34.6"E	37 m	10	SEO0026 SKK
TEA	Teaha, Teaha-ri, Seo-myeon, Ulleung-eup, Ulleung-gun, Gyeongsangbuk-do	37°30'46.2"N 130°47'53.3"E	18 m	10	SEO0033 SKK
TON	Tonggumi, Namseo-ri, Seo-myeon, Ulleung-eup, Ulleung-gun, Gyeongsangbuk-do	37°27'37.3"N 130°51'52.2"E	34 m	10	SEO0016 SKK
**Total**				**255**	

N/I: Number of individuals in population.

* Number in parenthesis: Number of individiduals included in phylogenetic analysis.

** Species/populations excluded from the population analysis.

**Fig 2:**
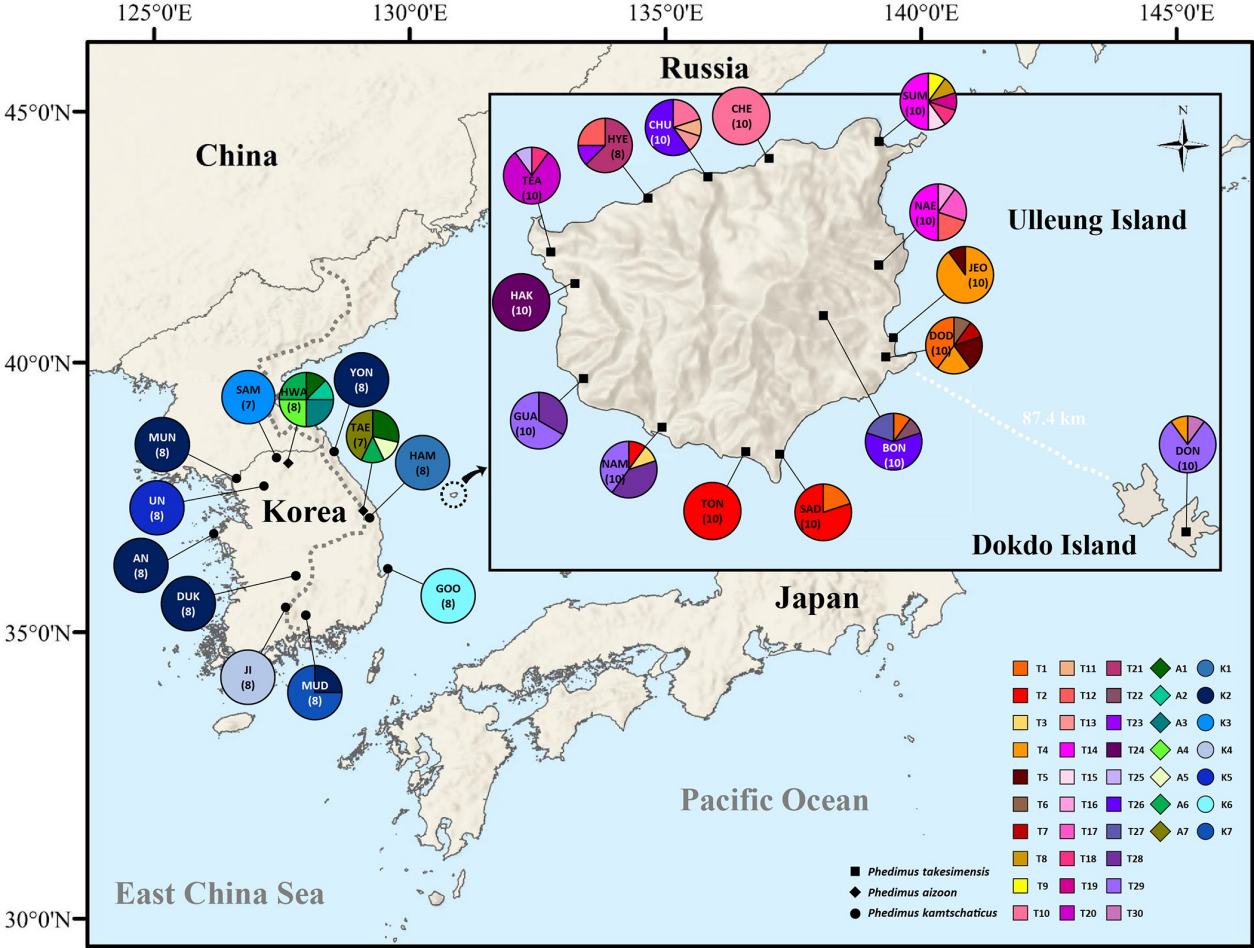
Map of the haplotypes found in insular derived *Phedimus takesimensis* and two continental progenitor species, *P*. *kamtschaticus* and *P*. *aizoon*. Different colored portions in each pie chart represent the haplotype frequencies.

For the population-level study of the continental progenitor and insular derivative species pairs, we sampled a total of two populations for *P*. *aizoon* (Table 1). In the case of the continental progenitor species *P*. *kamtschaticus*, we sampled a total of 10 populations (eight accessions per population, except for SAM) from Korea. *Phedimus kamtschaticus* occurs widely in East Asia, including eastern China, Russia, and Japan, but population-level sampling was not possible for those regions. The 10 populations from the Korean Peninsula represent the typical distribution range of *P*. *kamtschaticus*, which include the northeastern most population (YON), the southernmost population (MUD), and the westernmost population (AN), enabling us to assess their relationships to *P*. *takesimensis* (Fig 2 and Table 1). For the insular derived *P*. *takesimensis* species, which occurs exclusively on the seaside and sunny slopes of Ulleung Island and Dokdo Island, we sampled a total of 15 populations and 148 accessions; 14 populations were from Ulleung Island and one was from Dokdo Island, which is located about 87 km east of Ulleung Island. We sampled a total of 10 accessions per population (except for HYE with eight accessions), representing the northern region (HYE, CHU, and CHE), eastern region (SUM, NAE, JEO, DOD, and BON), southern region (DON, SAD, and TON), and southwestern/western region (NAM, GUA, HAK, and TEA) (Fig 2 and Table 1). Voucher specimens for the representative individuals of each population were deposited in the Ha Eun Herbarium (SKK), Sungkyunkwan University (Table 1).

### DNA isolation, amplification, and sequencing

Silica-gel dried leaves collected from the natural populations were used as DNA sources for the DNA extraction using DNeasy Plant mini kit (Qiagen, Carlsbad, California, USA) following the standard protocol. We screened 21 noncoding regions of chloroplast DNA and selected five variable noncoding regions (i.e., *atp*I-*atp*H, *trn*C^(GCA)^-*ycf*6, *trn*L-*trn*F, 3’*rps*16-5’*trn*K^(UUU)^, and *ndh*J-*trn*F^(GAA)^) [39]. We also amplified the nrDNA ITS region using the same primers described previously [40,41]. Polymerase chain reaction (PCR) amplification was conducted using the standard protocol of the Inclone Taq DNA polymerase kit (Inclone Biotech Co., Yongin, Korea) with a final volume of 50 μl consisting of 1 μl purified DNA, 5 μl reaction buffer, 1 μl dNTPs, 1 μl of each universal forward and reverse primer, 0.25 μl *Taq* polymerase, and 40.75 μl of ddH_2_O. The following PCR conditions were used for the chloroplast DNA regions: an initial denaturation of 95°C for 2 min, 35 cycles of 95°C denaturation for 1 min, 50–56°C annealing for 2 min, 72°C extension for 2 min, and a final extension at 72°C for 10 min. The following were the nrDNA ITS region reaction conditions: an initial denaturation of 94°C for 1 min, 35 cycles of 94°C denaturation for 1 min, 54°C annealing for 2 min, 72°C extension for 2 min, and a final extension at 72°C for 10 min. The PCR products were visualized using electrophoresis on a 1% agarose gel, purified using an Inclone Gel and PCR purification kit (Inclone Biotech Co., Yongin, Korea), and sequenced using Big Dye Terminator Cycle Sequencing reagents (Applied Biosystems, Forest City, CA, USA) at Geno Tech. Corp. (Daejeon, Korea). For the sequencing reactions, we used the same primers as those used for the PCR.

### Phylogenetic analysis, network construction, and population structure

All sequences were edited and assembled using Sequencher 4.2.2 (Gene Codes, Ann Arbor, MI, USA) and Geneious Pro v R.8 (Biomatters, Ltd, New Zealand), and were deposited at GenBank with the accession numbers MK752203–MK752392. The sequences were aligned using Clustal X version 1.83 [42], with a final manual adjustment using MacClade [43]. Gaps were coded as simple binary characters [44] using the program SeqState 1.4.1 [45]. To determine the species relationships among *Phedimus* in Korea, we conducted a separate phylogenetic analysis for the nrDNA ITS and four chloroplast noncoding regions (*trn*C-*ycf*6, *atp*I-*atp*H, *trn*L-*trn*F, and *ndh*J-*trn*F). The maximum likelihood (ML) analysis was conducted using IQ-TREE v. 1.4.2. [46], with 1,000 replicate bootstrap (BS) analyses, based on the best-fit model for each data set: “TVM+F+G4” for combined cpDNA and “TIM3e+G4” for nrDNA ITS.

For the population level analysis of insular derived *P*. *takesimensis* and the purported continental progenitors *P*. *kamtschaticus* and *P*. *aizoon* in Korea, we concatenated four previously used chloroplast noncoding regions and one more highly variable region (*rps*16-*trn*K) to achieve more resolutions. We constructed a haplotype network using TCS version 1.21 [47]. Gaps were treated as missing data and the connection limit excluding homoplastic changes was set to 95%, in accordance with Hart and Sunday [48]. To determine the overall relationships among accessions of the three species, we conducted ML analysis using IQ-TREE v. 1.4.2. [46] with the best-fit model of “K3Pu+F+I” and an unrooted tree was drawn. The pairwise distance based on the Kimura 2-parameter method [49] was also calculated using PAUP* 4.0b10 [50].

Genetic variation between taxa, and among and within populations was evaluated by an AMOVA (analysis of molecular variance) using ARLEQUIN version 3.5 [51]. The AMOVA analyses were performed using four categories, (a) all samples from the three species, (b) the insular derivative species *P*. *takesimensis*, (c) the continental progenitor species *P*. *aizoon*, and (d) the continental progenitor species *P*. *kamtschaticus*. The significance levels of the AMOVA were evaluated using 1,023 permutations. A SAMOVA (spatial analysis of molecular variance; [52]) was used to characterize patterns of genetic structure across the species distribution. For the demographic history, mismatch distributions were calculated and tested against sudden demographic expansion [53] using Arlequin 3.5 [51] and DnaSP v. 5 [54]. Unimodal patterns are expected for recent sudden population expansions, while multimodal distributions are suggestive of demographic stability or multiple colonizations [55]. Tajima’s *D* [56] and Fu’s *F*_*S*_ [57] were calculated to test for evidence of range expansion. To validate the fit of the models, we used the sum of squared deviations (*SSD*; [58]) between observed and expected distributions and Harpending’s raggedness index values (*RI*; [59]) of the observed distribution. Significant positive Tajima’s *D* and Fu’s *F*_*S*_ values indicate no sudden expansion events.

## Results

### Phylogenetic relationships between *Phedimus takesimensis* and congeneric species in Korea

**Fig 3:**
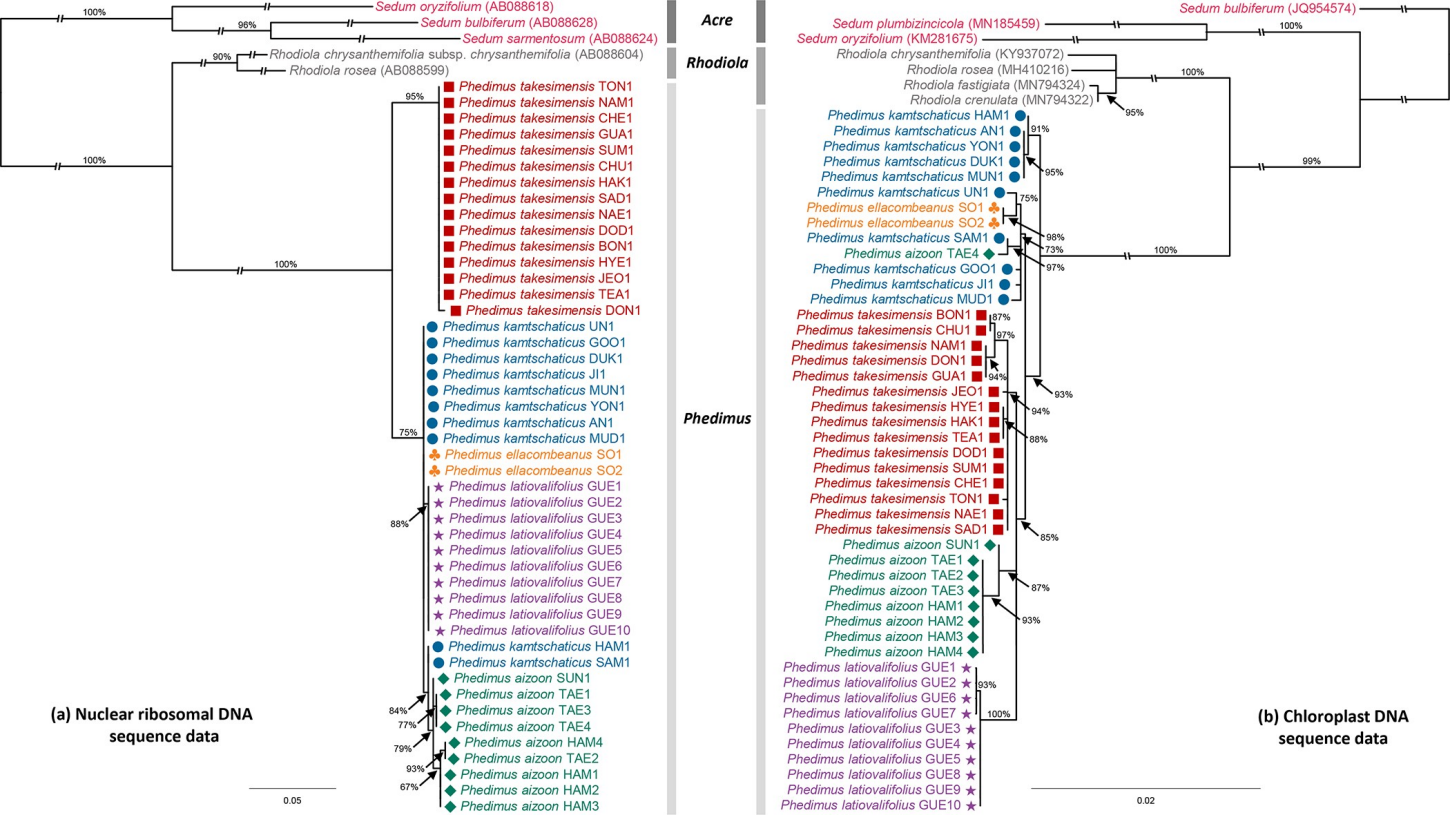
Maximum likelihood tree based on (a) nuclear ribosomal DNA internal transcribed spacer regions and (b) four chloroplast noncoding sequences. Bootstrap support values are shown at each node.

The total aligned length of the nrDNA ITS was 697 sites. ML analysis based on the nrDNA ITS showed that *Phedimus* was strongly supported as a monophyletic group (100%) (Fig 3A). It also identified two major lineages within the Korean representative species of *Phedimus*; one clade (95% BS) included *P*. *takesimensis* and the other (75% BS) included *P*. *kamtschaticus*, *P*. *ellacombeanus*, *P*. *latiovalifolius*, and *P*. *aizoon*. The latter lineage was not well resolved, but it showed that *P*. *ellacombeanus* is unresolved with *P*. *kamtschaticus*, while *P*. *latiovalifolius* (88% BS) and *P*. *aizoon* (79% BS) represent a separate distinct lineage. Two individuals of *P*. *kamtschaticus* (HAM1 and SAM1) were part of the *P*. *aizoon* clade (84%).

The total aligned sites of the four concatenated cpDNA noncoding regions and indel codings were 3,405 sites: *trn*L–*trn*F (1–758 sites), *atp*I–*atp*H (759–1,495 sites), *trn*C–*ycf*6 (1,496–2,288 sites), *ndh*J–*trn*F (2,289–3,289 sites), and indel codings (3,290–3,405 sites). ML analysis based on cpDNA showed that *Phedimus* was monophyletic (100% BS) (Fig 3B). The Ulleung Island endemic *P*. *takesimensis* and the other narrow Korean endemic *P*. *latiovalifolius* were also monophyletic, with 94% and 100% BS, respectively. All but one individual (TAE4) of *P*. *aizoon* formed a monophyletic group (87% BS), while *P*. *kamtschaticus* was highly polyphyletic. *Phedimus ellacombeanus* was monophyletic (98%) and deeply embedded within one lineage of *P*. *kamtschaticus* (73%). The ML tree based on the combined ITS and cpDNA datasets (not shown) was nearly identical to the cpDNA tree.

**Fig 4:**
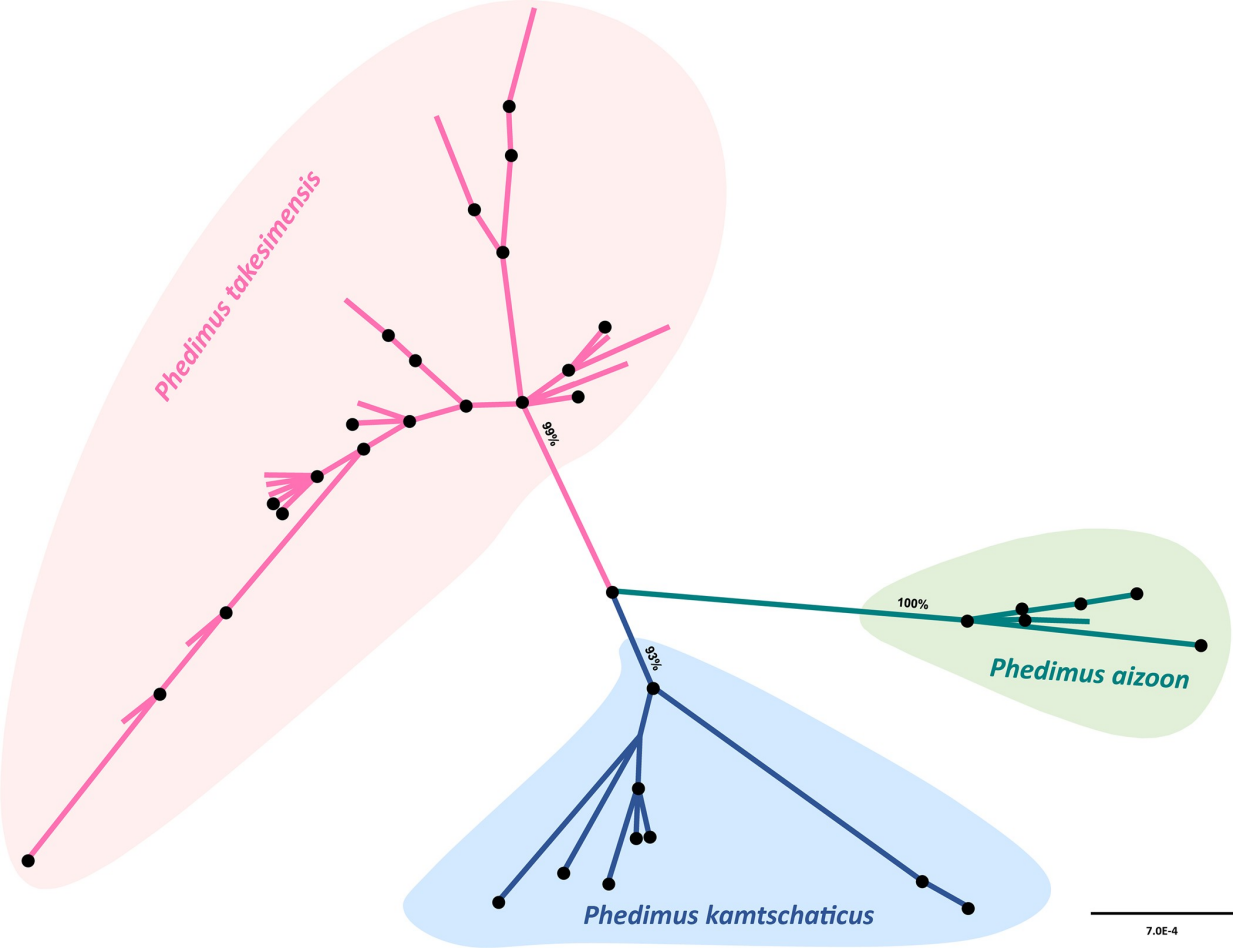
Unrooted maximum likelihood tree of insular derived *Phedimus takesimensis* and two continental progenitor species, *P*. *kamtschaticus* and *P*. *aizoon*. Bootstrap supports for the three species are shown.

We also assessed the relationships among three species, two purported continental progenitor species (*P*. *aizoon* and *P*. *kamtschaticus*) and insular derived *P*. *takesimensis*. The unrooted ML tree (Fig 4) showed that the three species all formed strongly supported groups; 99% BS for *P*. *takesimensis*, 100% for *P*. *aizoon*, and 93% for *P*. *kamtschaticus*. We also calculated the pairwise distance based on the Kimura 2-parameter method [49] within each species and among species. The average pairwise distance within *P*. *takesimensis* was 0.000878 ± 0.000564 (± standard deviation, SD), while that of *P*. *aizoon* and P. *kamtschaticus* was 0.000617 ± 0.0005 and 0.00174 ± 0.000131, respectively. In the case of the pairwise distances among species, between *P*. *takesimensis* and *P*. *aizoon* it was 0.003231 ± 0.0006, between *P*. *takesimensis* and *P*. *kamtschaticus* it was 0.003477 ± 0.0006, and between *P*. *aizoon* and *P*. *kamtschaticus* it was 0.00391 ± 0.0006.

### Chloroplast haplotype network and relationships among *Phedimus kamtschaticus*, *P*. *aizoon*, and *P*. *takesimensis*

The total length of five concatenated cpDNA noncoding regions and indel codings were 3,589 sites; *atp*I–*atp*H (1–662; 662 sites), *ndh*J–*trn*F (663–1,535; 873 sites), *rps*16–*trn*K (1,536–2,129; 594 sites), *trn*L–*trn*F (2,130–2,843; 714 sites), *trn*C–*ycf*6 (2,844–3,579; 736 sites), and indel codings (3,580–3,589; 10 sites). We found a total of 44 haplotypes, 30 for *P*. *takesimensis*, seven for *P*. *kamtschaticus*, and seven for *P*. *aizoon* (Table 2, Fig 2, and S1 Table). No sharing of haplotypes was found among the three species (Fig 2). All but one population (MUD) of *P*. *kamtschaticus* contained one haplotype, showing very low haplotype diversity within a population, while diverse haplotypes were found for *P*. *aizoon*, with five and four haplotypes for the HWA and TAE populations, respectively (Table 2 and Fig 2). For *P*. *takesimensis*, the number of haplotypes within a population ranged from one (CHE, HAK, and TON) to six (SUM) (Table 2). In addition, gene diversity and nucleotide diversity ranged from 0.0000 ± 0.0000 (CHE, HAK, and TON) to 0.8222 ± 0.0969 (DOD) and from 0.0000 ± 0.0000 (CHE and TON) to 0.002967 ± 0.001672 (CHU), respectively. One population (DON) on Dokdo Island contained three haplotypes (T4, T29, and T30), and showed gene diversity of 0.3778 ± 0.1813 and nucleotide diversity of 0.001561 ± 0.000927 (Table 2 and Fig 2). For the two populations of *P*. *aizoon*, much higher gene and nucleotide diversity were observed; 0.8929 ± 0.0858 and 0.000635 ± 0.000443 for HWA and 0.8095 ± 0.1298 and 0.000953 ± 0.000635 for TAE, respectively. Overall, *P*. *takesimensis* and *P*. *aizoon* showed similar levels of genetic diversity. In the case of nucleotide diversity, *P*. *takesimensis* and *P*. *kamtschaticus* are higher than *P*. *aizoon* (Table 3).

**Table 2:** Distribution of haplotypes (frequency in parentheses) and gene and nucleotide diversities among populations of insular derived *Phedimus takesimensis* and two putative continental progenitor species, *P*. *kamtschaticus* and *P*. *aizoon*.

Species	Code	Haplotype (Frequency)	No. of polymorphic sites	No. of observed indels	Gene diversity	Nucleotide diversity	N/I
*Phedimus takesimensis*	NAE	T12 (2) T14 (5) T16 (1) T17 (2)	24	19	0.7333 ± 0.1199	0.001521 ± 0.000905	10
	BON	T1 (1) T22 (1) T26 (6) T27 (2)	26	16	0.6444 ± 0.1518	0.002832 ± 0.001601	10
	CHE	T10 (10)	0	0	0.0000 ± 0.0000	0.000000 ± 0.000000	10
	CHU	T10 (2) T11 (1) T13 (1) T26 (6)	24	17	0.6444 ± 0.1518	0.002967 ± 0.001672	10
	DOD	T1 (4) T4 (2) T5 (2) T6 (1) T7 (1)	12	7	0.8222 ± 0.0969	0.001122 ± 0.000693	10
	DON	T4 (1) T29 (8) T30 (1)	27	19	0.3778 ± 0.1813	0.001561 ± 0.000927	10
	GUA	T28 (4) T29 (6)	1	0	0.5333 ± 0.0947	0.000150 ± 0.000153	10
	HAK	T24 (10)	0	0	0.0000 ± 0.0000	0.0000 ± 0.0000	10
	HYE	T12 (2) T21 (5) T23 (1)	13	4	0.6071 ± 0.1640	0.001117 ± 0.000711	8
	JEO	T4 (9) T5 (1)	8	7	0.2000 ± 0.1541	0.000451 ± 0.000330	10
	NAM	T2 (1) T3 (1) T28 (4) T29 (4)	25	17	0.7333 ± 0.1005	0.002242 ± 0.001288	10
	SAD	T1 (2) T2 (8)	6	5	0.3556 ± 0.1591	0.000602 ± 0.000412	10
	SUM	T8 (1) T9 (1) T14 (5) T15 (1) T18 (1) T19 (1)	16	6	0.7778 ± 0.1374	0.001032 ± 0.000645	10
	TEA	T18 (1) T20 (8) T25 (1)	4	0	0.3778 ± 0.1813	0.000226 ± 0.000200	10
	TON	T2 (10)	0	0	0.0000 ± 0.0000	0.0000 ± 0.0000	10
*Phedimus kamtschaticus*	AN	K2 (8)	0	0	0.0000 ± 0.0000	0.0000 ± 0.0000	8
	DUK	K2 (8)	0	0	0.0000 ± 0.0000	0.0000 ± 0.0000	8
	GOO	K6 (8)	0	0	0.0000 ± 0.0000	0.0000 ± 0.0000	8
	HAM	K1 (8)	0	0	0.0000 ± 0.0000	0.0000 ± 0.0000	8
	JI	K4 (8)	0	0	0.0000 ± 0.0000	0.0000 ± 0.0000	8
	MUD	K2 (2) K7 (6)	16	5	0.0000 ± 0.0000	0.0000 ± 0.0000	8
	MUN	K2 (8)	0	0	0.0000 ± 0.0000	0.0000 ± 0.0000	8
	SAM	K3 (7)	0	0	0.0000 ± 0.0000	0.0000 ± 0.0000	7
	UN	K5 (8)	0	0	0.0000 ± 0.0000	0.0000 ± 0.0000	8
	YON	K2 (8)	0	0	0.0000 ± 0.0000	0.0000 ± 0.0000	8
*Phedimus aizoon*	HWA	A1 (1) A2 (1) A3 (2) A4 (2) A6 (2)	4	1	0.8929 ± 0.0858	0.000635 ± 0.000443	8
	TAE	A1 (2) A5 (1) A6 (1) A7 (3)	6	1	0.8095 ± 0.1298	0.000953 ± 0.000635	7
		Total: 44	Mean: 3.259				

N/I: Number of individuals in population. Codes representing different populations are described in Table 1.

**Table 3:** Summary of chloroplast DNA variation for *Phedimus takesimensis*, *P*. *aizoon*, and *P*. *kamtschaticus*.

No. of haplotypes	No. of polymorphic sites	No. of observed transitions	No. of observed transversions	No. of substitutions	No. of indels	Gene diversity	Nucleotide diversity (*π*)
***Phedimus takesimensis* (15 populations; 148 individuals)**							
30	82	9	23	32	52	0.9346 ± 0.0068	0.003290 ± 0.001658
***Phedimus aizoon* (two populations; 15 individuals)**							
7	7	3	4	7	1	0.8952 ± 0.0433	0.000905 ± 0.000555
***Phedimus kamtschaticus* (10 populations; 79 individuals)**							
7	25	10	10	20	5	0.7699 ± 0.0378	0.001971 ± 0.001037
**Total: 40**	Mean: 38	Mean: 7.333	Mean: 12.333	Mean: 19.667	Mean: 19.333		

**Fig 5:**
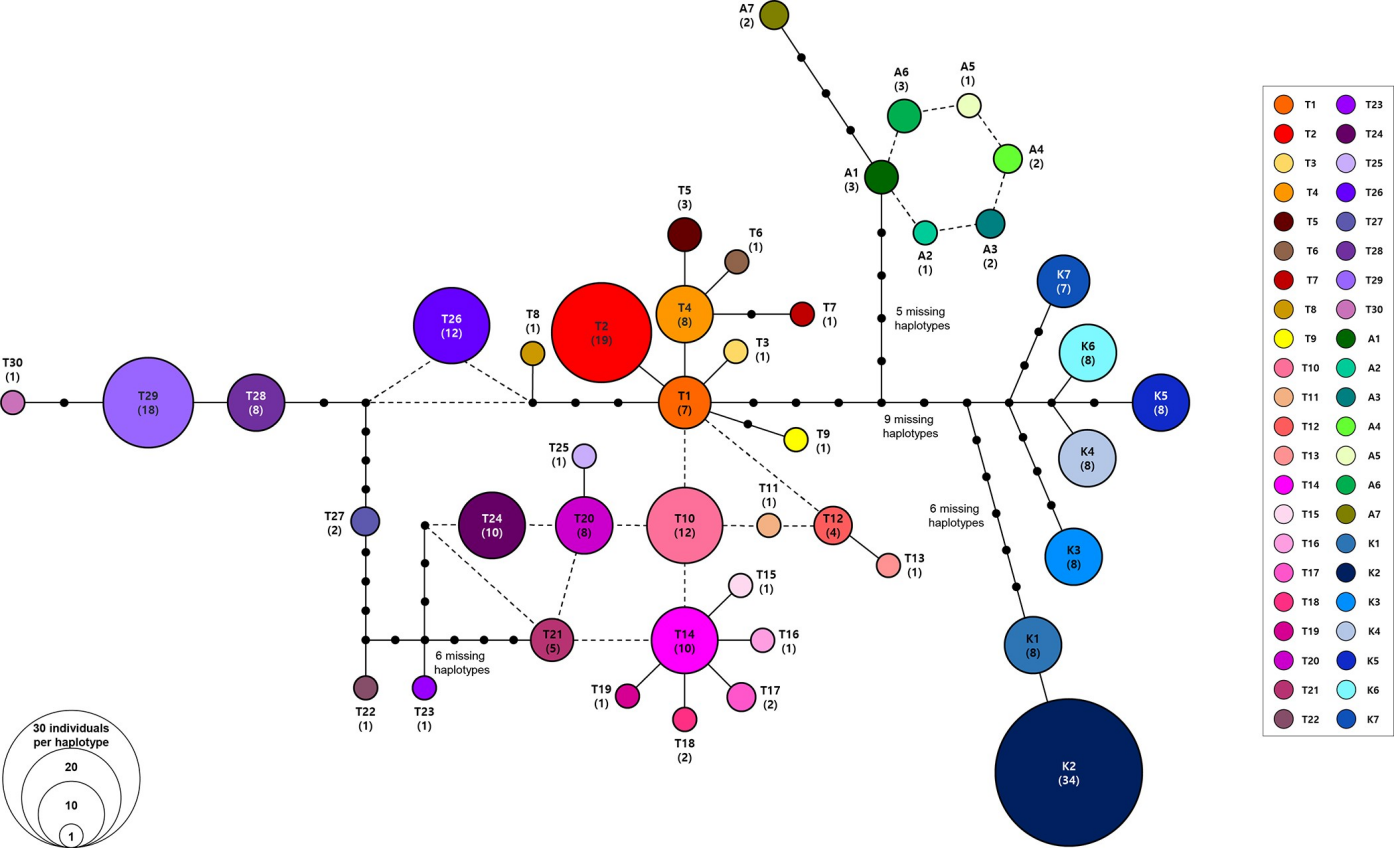
TCS haplotype network. Relationships among the 44 haplotypes found in *Phedimus takesimensis* on Ulleung Island and the two continental progenitor species, *P*. *kamtschaticus* and *P*. *aizoon*. Small dots represent either missing or inferred haplotypes and the size of each circle is proportional to the population size.

In terms of haplotype relationships, haplotypes A1–A6 of *P*. *aizoon* formed a ring-like network structure, and haplotype A7 was distantly related to the ring structure by three missing haplotypes (Fig 5). Despite much broader sampling of *P*. *kamtchaticus* compared to *P*. *aizoon* in the Korean Peninsula, we found only seven somewhat divergent haplotypes in *P*. *kamtchaticus*. The haplotype K2 was the most common haplotype (34 accessions), while the remaining haplotypes showed similar frequency levels (Fig 5). The second most common haplotype was T2 (19 accessions), followed by T29 (18 accessions), T26 (12 accessions), and T10 (12 accessions). Several haplotypes (e.g., T3, T6, T7, etc.) were represented by just a single accession. We found a total of three haplotypes in the Dokdo Island population (DON). Of these three haplotypes, two (T4 and T29) were shared with Ulleung Island populations (DOD and JEO for T4, and GUA and NAM for T29), while the other T30 haplotype was unique to Dokdo Island, derived by one mutational step from the T29 haplotype (Table 2 and Figs 2 and 5).

### Genetic structure and demographic change

Although only two populations were sampled for *P*. *aizoon*, we estimated genetic variation among and within the populations by AMOVA. We conducted the AMOVA using four categories, (a) all samples from the three species, (b) *P*. *takesimensis*, (c) *P*. *aizoon*, and (d) *P*. *kamtschaticus*. When all samples from the three species were analyzed, 62% of the variation was found among the three taxa, followed by among populations (27.9%) and within populations (9.78%) (Table 4). In the case of continental progenitor species *P*. *kamtschaticus*, most of the variation was found among populations (90.84%), with 9.16% of the variation within populations. In contrast, 77% of the variation was found within populations and approximately 23% among populations in *P*. *aizoon*. In the case of the anagenetically derived insular *P*. *takesimensis*, around 70% of the variation was found among populations and 30% was found within populations.

**Table 4:** Summary of the analysis of molecular variance (AMOVA) for genetic differences between three *Phedimus* species (*P*. *takesimensis*, *P*. *aizoon*, and *P*. *kamtschaticus*).

Taxon	Source of variation	*d*.*f*.	Sum of squares	Variance components	Percentage variation (%)	Fixation indices	P-value
**(a) *P*. *takesimensis*, *P*. *aizoon* & *P*. *kamtschaticus***	Between taxa	2	1142.109	8.62935	62.32	*F*_CT:_ 0.62318	<0.001± 0.00000
	Among populations	24	869.511	3.86311	27.90	*F*_SC:_ 0.74035	<0.001 ± 0.00000
	Within populations	215	291.293	1.35485	9.78	*F*_ST:_ 0.90216	<0.001 ± 0.00000
	Total	241	2302.913	13.84732	100		
**(b) *P*. *takesimensis***	Among populations	14	616.657	4.27504	69.52	*F*_ST:_ 0.69521	<0.001 ± 0.00000
	Within Populations	133	249.275	1.87425	30.48		
	Total	147	865.932	6.14929	300		
**(c) *P*. *aizoon***	Among populations	1	4.449	0.41020	22.84	*F*_ST:_ 0.22837	0.03421 ± 0.00592
	Within Populations	13	18.018	1.38599	77.16		
	Total	14	22.467	1.79619	700		
**(d) *P*. *kamtschaticus***	Among populations	9	248.405	3.45027	90.84	*F*_ST:_ 0.90842	<0.001 ± 0.00000
	Within Populations	69	24.000	0.34783	9.16		
	Total	78	272.405	3.79809	1500		

Degrees of freedom (*d*.*f*.), sum of squares (SS), variance components and the percentage of variation (%) and its associated significance (*n* = 1,023 permutations) for the analyses are shown.

F_CT_: Proportion of genetic variation among groups.

F_SC_: Proportion of genetic variation between populations within groups.

F_ST_: Proportion of genetic variation between populations and groups overall

**Fig 6:**
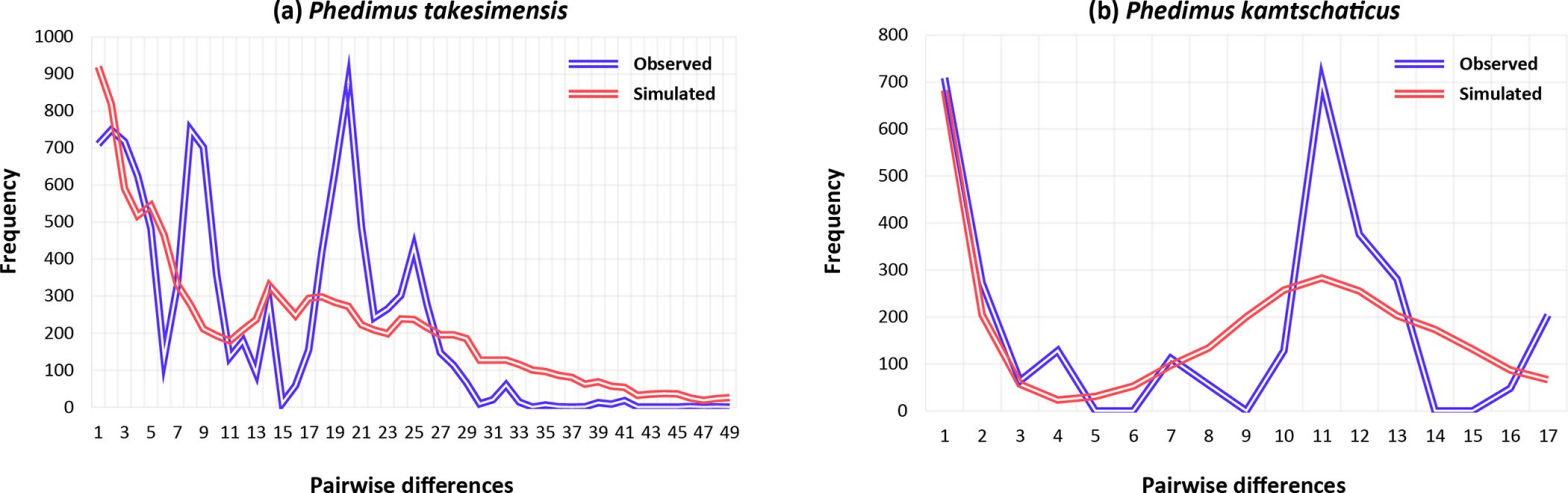
Mismatch analysis. Mismatch distribution analysis inferring the demographic history of *Phedimus takesimensis* (a) and *P*. *kamtschaticus* (b). The x-axis represents the number of pairwise differences, while the y-axis represents the relative frequency of pairwise comparisons.

**Fig 7:**
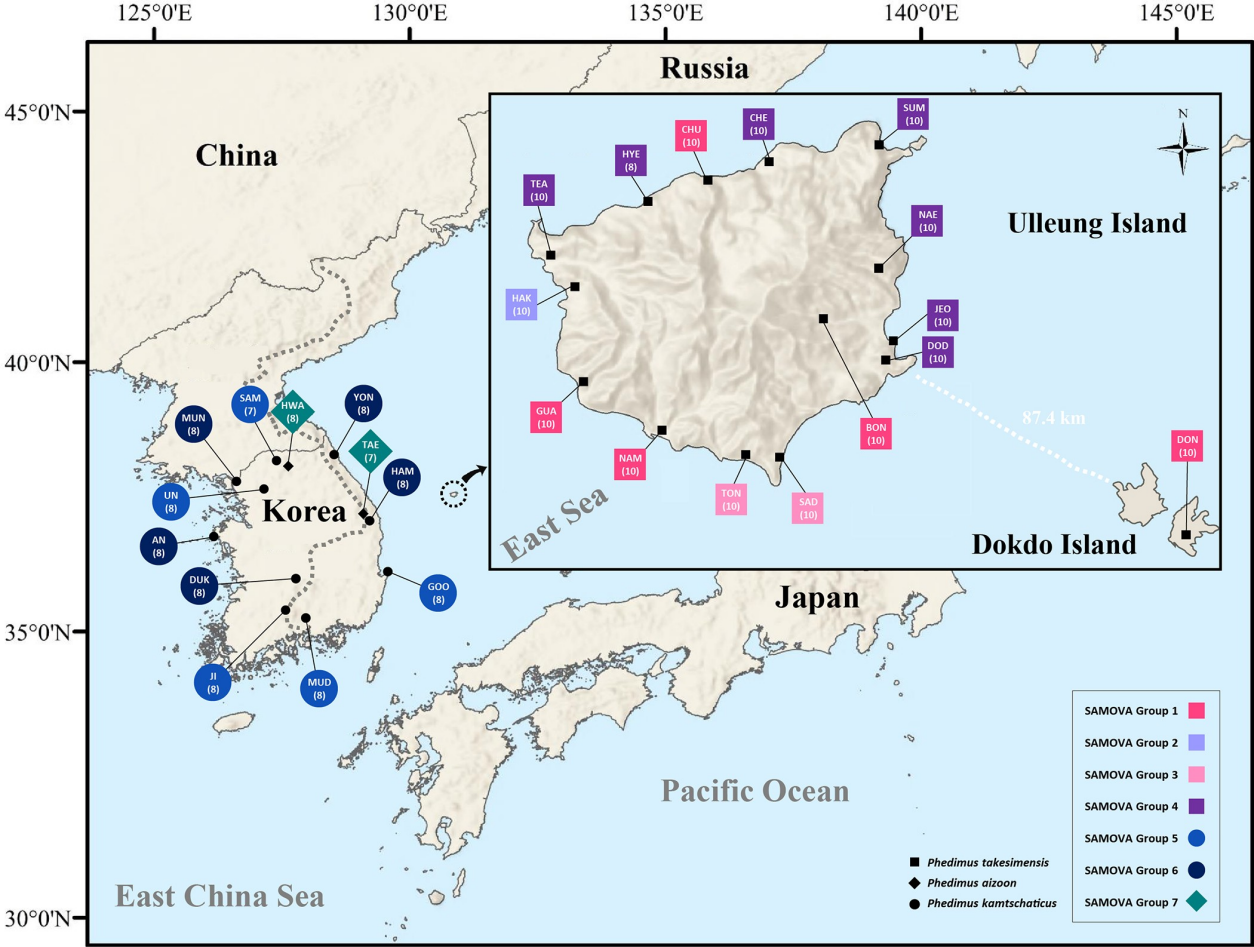
Spatial analysis of molecular variance (SAMOVA) grouping. Shapes and colors represent the different groups used in the SAMOVA analysis. Groups 1–4 represent *Phedimus takesimensis* on Ulleung Island. The two continental progenitor species are represented by Groups 5 and 6 (*P*. *kamtschaticus*) and Group 7 (*P*. *aizoon*).

Mismatch distributions showed that two species, *P*. *takesimensis* and *P*. *kamtschaticus*, had multimodal distributions, suggesting constant population sizes, multiple colonization, and/or sustained subdivision for a long period of time (Fig 6). In addition, Tajima’s *D* and Fu’s *F*_*S*_, and Ramos-Onisins and Rozas’ *R*_*2*_ failed to detect significant population expansion in the two species (Table 5). In the case of the SAMOVA analysis, we detected seven groups (*F*_CT_ = 0.79116) as the optimal number of genetic groups (*K*) based on spatial locations and cpDNA haplotypes (Table 6). The first four SAMOVA groups (Groups 1–4) included populations of *P*. *takesimensis* on Ulleung and Dokdo Islands, whereas the remaining three groups exclusively occurred on the Korean Peninsula (Fig 7). Group 1 contained five populations of *P*. *takesimensis*, four (CHU, GUA, NAM, and BON) on Ulleung Island and one (DON) on Dokdo Island (Fig 6). Group 2 included one population (HAK) of *P*. *takesimensis* from the northwestern part of Ulleung Island. Group 3 included two geographically close populations (TON and SAD) from the south-central part of Ulleung Island, while Group 4 included populations from the north facing (TEA, HYE, CHE, and SUM) and east facing (NAE, JEO, and DOD) part of the island. In the case of the three SAMOVA groups from the Korean Peninsula, Group 5 contained one central (UN), one northern (SAM), and three southern (GOO, JI, and MUD) populations of *P*. *kamtschaticus*, while Group 6 generally contained eastern (YON and HAM) and western (MUN, AN, and DUK) parts of the *P*. *kamtschaticus* populations. Group 7 exclusively contained two populations (HWA and TAE) of *P*. *aizoon*.

**Table 5:** Neutrality and population expansion tests for *Phedimus takesimensis* and *P*. *kamtschaticus*.

Statistics	*Phedimus takesimensis*	*Phedimus kamtschaticus*	Mean	s.d.
Tajima’s *D* test	-0.53610	1.64096	0.55243	1.53942
Tajima’s *D P*-value	0.34100 ^ns^	0.95900 ^ns^	0.65000	0.43699
Fu’s *F*s test	0.58420	9.79169	5.18794	6.51068
Fu’s *F*s *P*-value	0.63800 ^ns^	0.97400 ^ns^	0.80600	0.23759
Sum of Squared deviation (*SSD*)	0.01155	0.04045	0.02600	0.02044
SSD *P*-value	0.82000 ^ns^	0.36000 ^ns^	0.59000	0.32527
Raggedness index (*RI*)	0.00946	0.09325	0.05135	0.05925
Raggedness *P*-value	0.87000 ^ns^	0.46000 ^ns^	0.66500	0.28991

Tajima’s *D*, Fu’s *F*s, Sum of Squared deviation (*SSD*), and Raggedness index (*RI*) with significance derived from 10,000 simulations.

ns: not significant (P > 0.05); s.d.: standard deviation.

**Table 6:** Comparison of the fixation indices corresponding to the groups of populations detected by the spatial analysis of molecular variance (SAMOVA) in *Phedimus takesimensis*, *P*. *kamtschaticus*, and *P*. *aizoon* based on five chloroplast DNA regions.

No. of groups (*K*)	Percentage of variation			Fixation indices		
	Among groups	Among populations within groups	Within populations	*F*_SC_	*F*_ST_	*F*_CT_
2	55.43%	34.66%	9.90%	0.77777	0.90096	0.55434
3	63.18%	25.95%	10.88%	0.70462	0.89124	0.63178
4	70.87%	18.20%	10.93%	0.62464	0.89065	0.70869
5	73.67%	14.97%	11.36%	0.56847	0.88636	0.73666
6	76.03%	12.58%	11.39%	0.52485	0.88609	0.76026
7	79.12%	8.81%	12.07%	0.42186	0.87926	0.79116
8	78.59%	9.34%	12.06%	0.43645	0.87937	0.78594
9	78.85%	8.83%	12.32%	0.41761	0.87683	0.78851
10	80.27%	7.50%	12.23%	0.37992	0.87766	0.8027

*F*_SC_: Proportion of genetic variation between populations within groups.

*F*_ST_: Proportion of genetic variation between populations and groups overall.

*F*_CT_: Proportion of genetic variation among groups.

## Discussion

In this study, we provide the first convincing evidence for the monophyly of *P*. *takesimensis* on Ulleung and Dokdo Islands, despite its morphological variations within the islands and geographical proximity to continental source areas [26]. Based on the extensive sampling of the islands and using chloroplast and nuclear DNA, there is conclusive evidence that a single colonization event is responsible for the morphological and molecular diversity of *P*. *takesimensis* on the islands (Fig 3). In addition, both nuclear (ITS; 95% BS) and chloroplast (four noncoding regions; 94% BS) phylogenies strongly support the monophyly of *P*. *takesimensis*. This finding contrasts with other Ulleung Island endemics with multiple origins, such as *Rubus takesimensis* (Rosaceae; [60,61]) and *Scrophularia takesimensis* (Scrophulariaceae; [62]). However, the single origin of *P*. *takesimensis* is in line with other anagenetically derived endemics, such as *Dystaenia takesimana* (Apiaceae; [63]), *Hepatica maxima* (Ranunculaceae; [64]), *Acer okamotoanum* (Sapindaceae; [65]), *A*. *takesimense* (Sapindaceae; [66]), *Fagus multinervus* (Fagaceae; [67]), and *Campanula takesimana* (Campanulaceae; [68]). Therefore, despite the geographical proximity of Ulleung Island to possible source areas, a single origin for anagenetically originated endemics is the norm, with few exceptions.

Although we established the single origin of *P*. *takesimensis* on the Ulleung and Dokdo Islands, the closest sister species on the continents and geographical source areas are yet to be determined. While the nuclear ITS phylogeny suggested a sister relationship of monophyletic *P*. *takesimensis* to the clade containing *P*. *kamtschaticus*, *P*. *ellacombianus*, *P*. *latiovalifolius*, and *P*. *aizoon*. (Fig 3A), the cpDNA phylogeny suggested that *P*. *takesimensis* was unresolved with species of *P*. *aizoon* and *P*. *latiovalifolius* (Fig 3B). This unresolved relationship was also suggested based on nearly equal genetic distances of *P*. *takesimensis* to either *P*. *aizoon* or *P*. *kamtschaticus* (Fig 4) and the haplotype network (Fig 5). Although full picture of *Phedimus* phylogeny is yet to be revealed, it seems highly likely that the conflicts between nuclear and plastid phylogeny are due to hybridization and introgression among the sampled species in the current study. In addition, aneuploidization and polyploidization, which are known to occur commonly in genus *Phedimus* and related genera, likely contributed to such phylogenetic incongruences [32–35]. The important role of hybridization and aneuploidization/polyploidization in the evolution of *Phedimus* and related genera is yet to be investigated. It is not feasible to identify source areas for reciprocally monophyletic continental progenitors and insular derived species pairs (e.g., *P*. *kamtschaticus*/*P*. *aizoon* with *P*. *takesimensis*, *A*. *pseudosieboldianum* with *A*. *takesimense*, *A*. *mono* with *A*. *okamotoanum*, *Dystaenia ibukiensis* with *D*. *takesimana*, and *Fagus japonica*/*F*. *engleriana* with *F*. *multivervis*). However, given that the monophyletic insular endemics were deeply embedded within the paraphyletic continental species relationships, it was possible to determine the continental source populations for certain taxa, e.g., the origin of *Hepatica maxima* from the eastern Korean Peninsula population of *H*. *asiatica* [64] and the origin of *Campanula takesimana* from the southeastern Korean Peninsula population of *C*. *punctata* [68]. In the case of multiple origins of *Rubus takesimensis*, it was shown that the northern mainland Korean Peninsula and southern Korean Peninsula/Japanese archipelago populations were responsible for its origin on Ulleung Island [61].

As we described in the Introduction, *P*. *kamtschaticus* is one of most likely continental progenitor species of anagenetically derived endemic *P*. *takesimensis* on Ulleung Island. Therefore, this study also provided an opportunity to assess the genetic consequences of anagenetic speciation [69,70]. Based on the emerging genetic patterns for anagenetically derived species on islands, we expected no significant reduction in genetic diversity compared to continental progenitor species and a lack of strong genetic differentiation for *P*. *takesimensis* on the island. Although our sampling between the two species pairs was uneven, it seems there is no apparent reduction in genetic diversity in the insular derived *P*. *takesimensis*. For example, *P*. *takesimensis* showed much higher gene diversity (0.9346) and nucleotide diversity (0.003290) compared to *P*. *kamtchaticus* (gene diversity of 0.7699 and nucleotide diversity of 0.00197) (Table 3). In addition, unlike the highly monomorphic haplotypes that exist within the population of the continental progenitor *P*. *kamtchaticus*, we found highly diverse haplotypes in *P*. *takesimensis* on Ulleung Island, with 30 and 7 haplotypes for *P*. *takesimensis* and *P*. *kamtchaticus*, respectively (Table 3 and Fig 5). These results, however, should be interpreted cautiously given the uneven sampling of progenitor and derivative species pairs. Nevertheless, these results are consistent with previously studied taxa, such as *Rubus takesimensis* [61], *Acer takesimense* and *A*. *okamotoanum* [65,66], and *Dystaenia takesimana* [63].

We also found that, based on the same molecular marker type of chloroplast DNA and the AMOVA results, a significant portion of the genetic variation existed between taxa (62%), suggesting genetic divergence among the three species (Table 4). Furthermore, genetic variations appeared to be partitioned primarily into geographical regions (SAMOVA results; Fig 7), although around 30% of the variation still existed within populations of *P*. *takesimensis*. When we compared these results to other endemics on Ulleung Island, we found 43% of the variation in *Rubus takesimensis* was within populations [61] and 48% was within populations in *Campanula takesimana* [68]. Therefore, *P*. *takesimensis* showed apparent population genetic structure and differentiation after its divergence from the continental progenitor species. Given the fruit type of *P*. *takesimensis* are aggregates of follicles, with the fruit opening along an upward facing suture (i.e., cup-shaped), and with small light-weighted seeds, it is highly conceivable that raindrops are responsible for splashing out and flushing away seeds over some distances [71–73]. This type of splash rain dispersal mechanism was documented for *Sedum formosanum* and *S*. *subtile* from the same family (Crassulaceae) in Japan and it was shown experimentally that the maximum seed dispersal distance of *S*. *subtile* was 63.7 ± 7.8 cm [73]. Therefore, it is highly likely that splash seed dispersal by raindrops is responsible for the limited seed-mediated gene flow of populations of *P*. *takesimensis*, which have apparent population genetic structure and differentiation.

In this study, we also demonstrated that the population of *P*. *takesimensis* on eastern Dokdo Island (i.e., the eastern islet) most likely originated from two separate populations, one in southwestern Ulleung Island (GUA and NAM) and the other in southeastern Ulleung Island (JEO and DOD) (Fig 2). Dokdo Island is located approximately 90 km away from the east of Ulleung Island, with an estimated age of 4.6–2.5 myr, and is much older than Ulleung Island (approximately 2 myr) [17,74]. One common haplotype found on Dokdo Island, T29, was also found in two southwestern parts of Ulleung Island, while one low frequency haplotype on Dokdo Island, T4, was also found in the southeastern part of Ulleung Island with a relatively high frequency, especially in JEO (nine accessions with the T4 haplotype). One rare haplotype, T30, was found exclusively on Dokdo Island and given its haplotype network relationship (Fig 5), it is highly likely that this unique haplotype originated after colonization from Ulleung Island, especially from the southwestern haplotype (T29). However, we cannot completely rule out the possibility of sampling error for the lack of this haplotype on Ulleung Island. In the case of *C*. *takesimana*, which occurs on both Ulleung and Dokdo (western islet) Islands, we demonstrated that Dokdo Island was the steppingstone island for its origin on Ulleung Island [68].

In conclusion, we provide the first convincing evidence, based on extensive sampling, that *P*. *takesimensis* on Ulleung Island is a strongly supported monophyletic group that is closely related to *P*. *aizoon* and *P*. *kamtschaticus* in the Korean Peninsula. We also found that *P*. *takesimensis* on Ulleung Island and *P*. *aizoon* on the peninsula harbor diverse haplotypes, while *P*. *kamtschaticus* shows highly monomorphic haplotypes. The overall genetic diversity parameters also suggested that insular derived *P*. *takesimensis* maintains a high genetic diversity compared to its continental progenitor species. Unlike other anagenetically originated endemics, *P*. *takesimensis* showed population genetic structuring and we suggest that limited seed-mediated gene flow via a splash rain dispersal mechanism is responsible for this genetic pattern. Lastly, we demonstrated that the Dokdo Island was colonized by two separate gene pools of *P*. *takesimensis* (southwestern and southeastern) from Ulleung Island.

## Supporting information

S1 Table Variable sites (substitutions and indels) found in *Phedimus takesimensis* (T1-T30), *P. kamtschaticus* (K1-K7), and *P. aizoon* (A1-A7), identifying 44 haplotypes. (DOCX)
