# Supplementary material for: Chloroplast DNA insights into the phylogenetic position and anagenetic speciation of Phedimus takesimensis (Crassulaceae) on Ulleung and Dokdo Islands, Korea
Source: PLoS One. 2020 Sep 28;15(9):e0239734. doi: 10.1371/journal.pone.0239734 (PMC7521733; doi:10.1371/journal.pone.0239734)
Supplement: S1 Table — (DOCX) [file pone.0239734.s001.docx]

***Supplementary Material***

**Chloroplast DNA insights into the phylogenetic position and anagenetic speciation of *Phedimus takesimensis* (Crassulaceae) on Ulleung and Dokdo Islands, Korea**

**Hee-Seung Seo^¶,#a^, Seon-Hee Kim^¶^, Seung-Chul Kim^*^**

**Correspondence: Seung-Chul Kim:** [**sonchus96@skku.edu**](mailto:sonchus96@skku.edu) **or sonchus2009@gmail.com**

**Supplementary Tables**

Supplementary Table 1. Variable sites (substitutions and indels) found in *Phedimus takesimensis* (T1-T30)*, P. kamtschaticus* (K1-K7), and *P. aizoon* (A1-A7), identifying 44 haplotypes.

| Haplotype | *trn*L^(UAA)^‐*trn*F^(GAA)^ | | | | | | | | | | | | | | *atp*I‐*atp*H | |
| --- | --- | --- | --- | --- | --- | --- | --- | --- | --- | --- | --- | --- | --- | --- | --- | --- |
|  | 28 | 224 | 289 | 306 | 363 | 405 | 435-439 | 486 | 548 | 552 | 565 | 574 | 683-688 | 756 | 823 | 1073 |
| T1 | A | A | C | C | C | A | AATTG | C | G | A | G | A | **------** | C | T | T |
| T2 | A | A | C | C | C | A | AATTG | C | G | A | G | A | **------** | C | T | T |
| T3 | G | A | C | C | C | A | AATTG | C | G | A | G | A | **------** | C | T | T |
| T4 | A | A | C | C | C | A | AATTG | C | G | A | G | A | **------** | C | T | T |
| T5 | A | A | C | C | C | A | AATTG | C | G | A | G | A | **------** | C | T | T |
| T6 | A | A | C | C | C | A | AATTG | C | G | A | G | A | **------** | C | T | T |
| T7 | A | A | C | C | C | A | AATTG | C | G | A | G | A | **------** | C | T | T |
| T8 | A | A | C | C | C | A | AATTG | C | G | C | G | A | ATTCAC | C | T | T |
| T9 | A | A | C | C | C | A | AATTG | C | G | A | G | A | **------** | C | T | T |
| T10 | A | A | C | C | C | A | AATTG | C | G | A | G | A | **------** | C | T | T |
| T11 | A | A | C | C | A | A | AATTG | C | G | A | G | A | **------** | C | T | T |
| T12 | A | A | C | C | A | A | AATTG | C | G | A | G | A | **------** | C | T | T |
| T13 | A | A | C | C | A | A | **-----** | C | G | A | G | A | **------** | C | T | T |
| T14 | A | A | C | C | C | A | AATTG | C | G | A | G | A | **------** | C | T | T |
| T15 | A | A | C | C | C | A | AATTG | C | G | A | G | A | **------** | C | T | T |
| T16 | A | A | C | C | C | A | AATTG | C | G | A | G | A | **------** | C | T | T |
| T17 | A | A | C | C | C | A | AATTG | C | G | A | G | A | **------** | C | T | T |
| T18 | A | A | C | C | C | A | AATTG | C | G | A | G | A | **------** | C | T | T |
| T19 | A | A | C | C | C | A | AATTG | C | G | A | G | A | **------** | C | T | T |
| T20 | A | C | C | C | C | A | AATTG | C | G | A | G | A | **------** | C | T | T |
| T21 | A | C | C | C | C | A | AATTG | C | G | A | G | A | **------** | C | T | T |
| T22 | A | A | C | C | C | A | AATTG | C | G | A | G | A | **------** | C | T | G |
| T23 | A | C | C | C | C | A | AATTG | C | G | A | G | A | **------** | C | T | T |
| T24 | A | C | C | C | C | A | AATTG | C | G | A | G | A | **------** | C | T | T |
| T25 | A | C | C | C | C | A | AATTG | C | G | A | G | A | **------** | C | T | T |
| T26 | A | A | C | C | C | A | AATTG | C | G | C | G | A | ATTCAC | C | T | T |
| T27 | A | A | C | C | C | A | AATTG | C | G | C | G | A | ATTCAC | C | T | T |
| T28 | A | A | C | T | C | A | AATTG | C | G | C | G | A | ATTCAC | C | T | T |
| T29 | A | A | C | T | C | A | AATTG | C | G | C | G | A | ATTCAC | C | T | T |
| T30 | A | A | C | T | C | A | AATTG | C | G | C | G | A | ATTCAC | C | T | T |
| K1 | A | A | T | C | C | A | AATTG | C | G | A | G | C | **------** | C | C | T |
| K2 | A | A | T | C | C | C | AATTG | C | G | A | G | C | **------** | C | C | T |
| K3 | A | A | T | C | C | A | AATTG | A | G | A | G | A | **------** | C | T | T |
| K4 | A | A | T | C | C | A | AATTG | C | G | A | G | A | **------** | C | T | T |
| K5 | A | A | T | C | C | A | AATTG | C | A | A | G | A | **------** | C | T | T |
| K6 | A | A | T | C | C | A | AATTG | C | G | A | G | A | **------** | C | T | T |
| K7 | A | A | T | C | C | A | AATTG | C | G | A | G | A | **------** | C | T | T |
| A1 | A | A | T | C | C | A | AATTG | C | G | A | A | A | **------** | A | T | T |
| A2 | A | A | T | C | C | A | AATTG | C | G | A | A | A | **------** | A | T | T |
| A3 | A | A | T | C | C | A | AATTG | C | G | A | A | A | **------** | A | T | T |
| A4 | A | A | T | C | C | A | AATTG | C | G | A | A | A | **------** | A | T | T |
| A5 | A | A | T | C | C | A | AATTG | C | G | A | A | A | **------** | A | T | T |
| A6 | A | A | T | C | C | A | AATTG | C | G | A | A | A | **------** | A | T | T |
| A7 | A | A | T | C | C | A | AATTG | C | G | A | A | A | **------** | A | T | T |

Continued.

| Haplotype | *atp*I‐*atp*H | | | | *trn*C^(GCA)^‐*ycf*6 | | | | | | | | | |
| --- | --- | --- | --- | --- | --- | --- | --- | --- | --- | --- | --- | --- | --- | --- |
|  | 1074-1078 | 1141 | 1190 | 1451 | 1535 | 1536 | 1537 | 1538 | 1539 | 1540 | 1553 | 1615 | 1628-1634 | 1697 |
| T1 | **-----** | G | C | A | T | A | G | T | G | T | G | A | CTCTAAA | A |
| T2 | **-----** | G | C | A | T | A | G | T | G | T | G | A | CTCTAAA | A |
| T3 | **-----** | G | C | A | T | A | G | T | G | T | G | A | CTCTAAA | A |
| T4 | **-----** | G | A | A | T | A | G | T | G | T | G | A | CTCTAAA | A |
| T5 | **-----** | G | A | A | T | A | G | T | G | T | G | A | CTCTAAA | A |
| T6 | **-----** | G | A | A | T | A | G | T | G | T | G | A | CTCTAAA | A |
| T7 | **-----** | G | A | A | T | A | G | T | G | T | G | A | CTCTAAA | A |
| T8 | **-----** | G | C | A | T | A | G | T | G | T | G | A | CTCTAAA | A |
| T9 | **-----** | G | C | A | T | A | G | T | G | T | G | A | CTCTAAA | A |
| T10 | **-----** | G | C | A | T | A | G | T | G | T | G | A | CTCTAAA | A |
| T11 | **-----** | G | C | A | T | A | G | T | G | T | G | A | CTCTAAA | A |
| T12 | **-----** | G | C | A | T | A | G | T | G | T | G | A | CTCTAAA | A |
| T13 | **-----** | G | C | A | T | A | G | T | G | T | G | A | CTCTAAA | A |
| T14 | **-----** | G | C | A | T | A | G | T | G | T | G | A | CTCTAAA | A |
| T15 | **-----** | G | C | A | T | A | G | T | G | T | G | A | CTCTAAA | A |
| T16 | **-----** | G | C | A | T | A | G | T | G | T | G | A | CTCTAAA | A |
| T17 | **-----** | G | C | A | T | A | G | T | G | T | G | A | CTCTAAA | A |
| T18 | **-----** | G | C | A | T | A | G | T | G | T | G | A | CTCTAAA | A |
| T19 | **-----** | G | C | A | G | A | G | T | G | T | G | A | CTCTAAA | A |
| T20 | **-----** | G | C | A | T | A | G | T | G | T | G | A | CTCTAAA | A |
| T21 | **-----** | G | C | A | T | A | G | T | G | T | G | A | CTCTAAA | A |
| T22 | **-----** | G | C | A | T | A | G | T | G | T | G | A | CTCTAAA | A |
| T23 | **-----** | G | C | A | T | A | G | T | G | T | G | A | CTCTAAA | A |
| T24 | **-----** | G | C | A | T | A | G | T | G | T | G | A | CTCTAAA | A |
| T25 | **-----** | G | C | A | T | A | G | T | G | T | G | A | CTCTAAA | A |
| T26 | **-----** | G | C | A | T | A | G | T | G | T | G | A | CTCTAAA | A |
| T27 | **-----** | G | C | A | T | A | G | T | G | T | G | A | CTCTAAA | A |
| T28 | **-----** | G | C | A | T | A | G | T | G | T | G | A | CTCTAAA | A |
| T29 | **-----** | G | C | A | T | A | G | T | G | T | G | A | CTCTAAA | A |
| T30 | **-----** | G | C | A | T | A | G | T | G | T | G | A | CTCTAAA | A |
| K1 | **-----** | G | C | G | T | A | G | T | G | T | G | G | **-------** | A |
| K2 | **-----** | G | C | G | T | A | G | T | G | T | G | G | **-------** | A |
| K3 | **-----** | C | C | A | T | A | G | T | G | T | G | A | **-------** | A |
| K4 | **-----** | G | C | A | T | A | G | T | G | T | G | A | **-------** | A |
| K5 | **-----** | G | C | A | T | A | G | T | G | T | G | A | **-------** | A |
| K6 | **-----** | G | C | A | T | A | G | T | G | T | G | A | **-------** | A |
| K7 | AAATT | G | C | A | T | A | G | T | G | T | G | A | **-------** | C |
| A1 | **-----** | G | C | A | T | A | G | T | G | T | C | A | **-------** | A |
| A2 | **-----** | G | C | A | T | A | G | G | G | T | C | A | **-------** | A |
| A3 | **-----** | G | C | A | T | A | T | G | G | T | C | A | **-------** | A |
| A4 | **-----** | G | C | A | T | A | T | G | G | T | C | A | **-------** | A |
| A5 | **-----** | G | C | A | T | A | T | T | G | T | C | A | **-------** | A |
| A6 | **-----** | G | C | A | T | A | G | T | G | T | C | A | **-------** | A |
| A7 | **-----** | G | C | A | T | G | A | T | C | C | C | A | **-------** | A |

Continued.

| Haplotype | *trn*C^(GCA)^‐*ycf*6 | | | | | | | | | | | | | |
| --- | --- | --- | --- | --- | --- | --- | --- | --- | --- | --- | --- | --- | --- | --- |
|  | 1783 | 1791 | 1830-1836 | 1846 | 1848 | 1850 | 1852 | 1853 | 1918 | 1956 | 1966 | 1968 | 1968 | 1985 |
| T1 | A | C | **-------** | A | A | A | T | T | A | G | G | A | A | G |
| T2 | A | C | **-------** | A | A | A | T | T | A | G | G | A | A | G |
| T3 | A | C | **-------** | A | A | A | T | T | A | G | G | A | A | G |
| T4 | A | C | **-------** | A | A | A | T | T | A | G | G | A | A | G |
| T5 | A | C | **-------** | A | A | A | T | T | A | G | G | A | A | G |
| T6 | A | C | **-------** | A | A | A | T | T | A | G | G | A | A | G |
| T7 | A | C | **-------** | A | A | A | T | T | G | G | G | A | A | G |
| T8 | A | C | **-------** | A | A | A | T | T | A | G | G | A | A | G |
| T9 | A | C | **-------** | A | A | A | A | T | A | G | G | A | A | G |
| T10 | A | C | **-------** | A | A | A | T | T | A | G | G | A | A | G |
| T11 | A | C | **-------** | A | A | A | T | T | A | G | G | A | A | G |
| T12 | A | C | **-------** | A | A | A | T | T | A | G | G | A | A | G |
| T13 | A | C | **-------** | A | A | A | T | T | A | G | G | A | A | G |
| T14 | A | C | **-------** | A | A | T | T | T | A | G | G | A | A | G |
| T15 | A | C | **-------** | A | A | T | T | T | A | G | G | A | A | G |
| T16 | A | C | **-------** | A | A | T | T | T | A | G | G | A | A | G |
| T17 | A | C | **-------** | A | A | T | T | T | A | A | G | A | A | G |
| T18 | A | C | **-------** | A | A | T | T | T | A | G | G | C | C | G |
| T19 | A | C | **-------** | A | A | T | T | T | A | G | G | A | A | G |
| T20 | A | C | **-------** | A | A | A | T | T | A | G | G | A | A | G |
| T21 | A | C | **-------** | A | A | T | T | T | A | G | G | A | A | G |
| T22 | A | C | TATA*---* | A | T | T | C | C | A | G | G | A | A | G |
| T23 | A | C | TATA**---** | T | T | T | C | C | A | G | G | A | A | G |
| T24 | A | C | TATA**---** | A | A | A | T | T | A | G | G | A | A | G |
| T25 | A | C | **-------** | A | A | A | T | T | A | G | G | A | A | G |
| T26 | A | C | **-------** | A | A | A | T | T | A | G | G | A | A | G |
| T27 | A | C | TATA**---** | A | T | T | C | C | A | G | G | A | A | G |
| T28 | A | C | **-------** | A | A | A | T | T | A | G | G | A | A | G |
| T29 | C | C | **-------** | A | A | A | T | T | A | G | G | A | A | G |
| T30 | C | C | **-------** | A | A | A | T | T | A | G | G | A | A | G |
| K1 | A | C | **-------** | A | A | A | T | T | A | G | G | A | A | T |
| K2 | A | C | **-------** | A | A | A | T | T | A | G | G | A | A | T |
| K3 | A | C | **-------** | A | A | A | T | T | A | G | A | A | A | T |
| K4 | A | A | **-------** | A | A | A | T | T | A | G | G | A | A | T |
| K5 | A | A | **-------** | A | A | A | T | T | A | G | G | A | A | T |
| K6 | A | A | **-------** | A | A | A | T | T | A | G | G | A | A | T |
| K7 | A | C | **-------** | A | A | A | T | T | A | G | G | A | A | T |
| A1 | A | C | TATATAT | A | A | A | T | T | A | G | G | A | A | T |
| A2 | A | C | TATATAT | A | A | A | T | T | A | G | G | A | A | T |
| A3 | A | C | TATATAT | A | A | A | T | T | A | G | G | A | A | T |
| A4 | A | C | TATATAT | A | A | A | T | T | A | G | G | A | A | T |
| A5 | A | C | TATATAT | A | A | A | T | T | A | G | G | A | A | T |
| A6 | A | C | TATATAT | A | A | A | T | T | A | G | G | A | A | T |
| A7 | A | C | TATATAT | A | A | A | T | T | A | G | G | A | A | T |

Continued.

| Haplotype | *trn*C ^(GCA)^‐*ycf*6 | | | | | *ndh*J‐*trn*F^(GAA)^ | | | | | |
| --- | --- | --- | --- | --- | --- | --- | --- | --- | --- | --- | --- |
|  | 2061 | 2093 | 2119 | 2213-2231 | 2276 | 2654 | 2700-2705 | 2772 | 2839 | 2887 | 2899-2905 |
| T1 | A | G | A | **-------------------** | C | C | **------** | C | T | C | CTCAAAA |
| T2 | A | G | A | **-------------------** | C | C | **------** | C | T | C | CTCAAAA |
| T3 | A | G | A | **-------------------** | C | C | **------** | C | T | C | CTCAAAA |
| T4 | A | G | A | **-------------------** | C | C | **------** | C | T | C | CTCAAAA |
| T5 | A | G | A | **-------------------** | C | C | **------** | C | T | C | **-------** |
| T6 | A | G | A | **-------------------** | C | C | **------** | C | T | C | CTCAAAA |
| T7 | A | G | A | **-------------------** | C | C | **------** | C | T | C | CTCAAAA |
| T8 | A | G | A | **-------------------** | C | C | **------** | C | T | C | CTCAAAA |
| T9 | A | G | A | **-------------------** | C | T | **------** | C | T | C | CTCAAAA |
| T10 | A | G | C | **-------------------** | C | C | **------** | C | T | C | CTCAAAA |
| T11 | A | G | A | **-------------------** | C | C | **------** | C | T | C | CTCAAAA |
| T12 | A | G | C | **-------------------** | C | C | **------** | C | T | C | CTCAAAA |
| T13 | A | G | A | **-------------------** | C | C | **------** | C | T | C | CTCAAAA |
| T14 | A | G | C | **-------------------** | C | C | **------** | C | T | C | CTCAAAA |
| T15 | A | G | C | **-------------------** | C | T | **------** | C | T | C | CTCAAAA |
| T16 | A | G | C | AGTTTACTATAGTTGCAAG | C | C | **------** | C | T | C | CTCAAAA |
| T17 | A | G | C | **-------------------** | C | C | **------** | C | T | C | CTCAAAA |
| T18 | A | G | C | **-------------------** | C | C | **------** | C | T | C | CTCAAAA |
| T19 | A | G | C | **-------------------** | C | C | **------** | C | T | C | CTCAAAA |
| T20 | A | G | C | **-------------------** | C | C | **------** | C | T | C | CTCAAAA |
| T21 | A | G | C | **-------------------** | C | C | **------** | C | T | C | CTCAAAA |
| T22 | A | G | A | **-------------------** | C | C | **------** | C | T | C | CTCAAAA |
| T23 | A | G | C | **-------------------** | C | C | **------** | C | T | C | CTCAAAA |
| T24 | A | G | C | **-------------------** | C | C | **------** | C | T | C | CTCAAAA |
| T25 | G | G | C | **-------------------** | C | C | **------** | C | T | C | CTCAAAA |
| T26 | A | G | A | **-------------------** | C | C | TTCACA | C | T | C | CTCAAAA |
| T27 | A | G | A | **-------------------** | C | C | TTCACA | C | T | C | CTCAAAA |
| T28 | A | G | A | **-------------------** | C | C | TTCACA | C | T | C | CTCAAAA |
| T29 | A | G | A | **-------------------** | C | C | TTCACA | C | T | C | CTCAAAA |
| T30 | A | G | A | **-------------------** | C | C | TTCACA | C | T | C | **-------** |
| K1 | A | G | A | **-------------------** | T | C | **------** | C | C | C | CTCAAAA |
| K2 | A | G | A | **-------------------** | T | C | **------** | C | C | C | CTCAAAA |
| K3 | A | G | A | **-------------------** | T | C | **------** | C | T | T | CTCAAAA |
| K4 | A | G | A | **-------------------** | T | C | **------** | C | T | T | CTCAAAA |
| K5 | A | G | A | **-------------------** | T | C | **------** | C | T | T | CTCAAAA |
| K6 | A | G | A | **-------------------** | T | C | **------** | C | T | T | CTCAAAA |
| K7 | A | T | A | **-------------------** | T | C | **------** | C | T | T | CTCAAAA |
| A1 | A | G | A | **-------------------** | C | C | **------** | A | T | C | CTCAAAA |
| A2 | A | G | A | **-------------------** | C | C | **------** | A | T | C | CTCAAAA |
| A3 | A | G | A | **-------------------** | C | C | **------** | A | T | C | CTCAAAA |
| A4 | A | G | A | **-------------------** | C | C | **------** | A | T | C | CTCAAAA |
| A5 | A | G | A | **-------------------** | C | C | **------** | A | T | C | CTCAAAA |
| A6 | A | G | A | **-------------------** | C | C | **------** | A | T | C | CTCAAAA |
| A7 | A | G | A | **-------------------** | C | C | **------** | A | T | C | CTCAAAA |

Continued.

| Haplotype | *ndh*J-*trn*L ^(GAA)^ | | | | | | | | | | | | |
| --- | --- | --- | --- | --- | --- | --- | --- | --- | --- | --- | --- | --- | --- |
|  | 2918 | 2934 | 2962 | 2964 | 3011-3015 | 3039 | 3217 | 3260 | 3266 | 3323 | 3422 | 3458 | 3515 |
| T1 | A | G | G | G | CTTAG | G | A | A | T | G | T | G | C |
| T2 | A | G | G | G | **-----** | G | A | A | T | G | T | G | C |
| T3 | A | G | G | G | CTTAG | G | A | A | T | G | T | G | C |
| T4 | A | G | G | G | CTTAG | G | A | A | T | G | T | G | C |
| T5 | A | G | G | G | CTTAG | G | A | A | T | G | T | G | C |
| T6 | A | G | G | G | CTTAG | G | A | A | T | T | T | G | C |
| T7 | A | G | G | G | CTTAG | G | A | A | G | G | T | G | C |
| T8 | T | G | G | G | CTTAG | G | A | A | A | G | T | G | C |
| T9 | A | G | G | G | CTTAG | G | A | A | T | G | T | G | C |
| T10 | A | G | G | G | CTTAG | G | A | A | T | G | T | G | C |
| T11 | A | G | G | G | CTTAG | G | A | A | T | G | T | G | C |
| T12 | A | G | G | G | CTTAG | G | A | A | T | G | T | G | C |
| T13 | A | G | G | G | CTTAG | G | A | A | T | G | T | G | C |
| T14 | A | G | G | G | CTTAG | G | A | A | T | G | T | G | C |
| T15 | A | G | G | G | CTTAG | G | A | A | T | G | T | G | C |
| T16 | A | G | G | G | CTTAG | G | A | A | T | G | T | G | C |
| T17 | A | G | G | G | CTTAG | G | A | A | T | G | T | G | C |
| T18 | A | G | G | G | CTTAG | G | A | A | T | G | T | G | C |
| T19 | A | G | G | G | CTTAG | G | A | A | T | G | T | G | C |
| T20 | A | G | G | G | CTTAG | G | A | A | T | G | T | G | C |
| T21 | A | G | G | G | CTTAG | G | A | A | T | G | T | G | C |
| T22 | A | G | G | G | CTTAG | G | A | A | T | G | T | G | C |
| T23 | A | G | G | G | CTTAG | G | A | A | T | G | T | G | C |
| T24 | A | G | G | G | CTTAG | G | A | A | T | G | T | G | C |
| T25 | A | G | G | G | CTTAG | G | A | A | T | G | T | G | C |
| T26 | T | G | G | G | CTTAG | G | A | A | T | G | T | G | C |
| T27 | T | G | G | G | CTTAG | G | A | A | T | G | T | G | C |
| T28 | A | G | G | G | CTTAG | G | A | A | G | G | T | G | C |
| T29 | A | G | G | G | CTTAG | G | A | A | G | G | T | G | C |
| T30 | A | G | G | G | CTTAG | G | A | A | T | G | T | G | C |
| K1 | A | G | G | A | CTTAG | G | G | C | T | G | T | G | C |
| K2 | A | G | G | A | CTTAG | G | G | C | T | G | T | G | C |
| K3 | A | G | G | A | CTTAG | G | A | C | T | G | T | G | A |
| K4 | A | G | G | A | CTTAG | G | A | C | T | G | C | G | C |
| K5 | A | G | T | A | CTTAG | G | A | C | T | G | T | G | C |
| K6 | A | G | G | A | CTTAG | A | A | C | T | G | T | G | C |
| K7 | A | G | G | A | CTTAG | G | A | C | T | G | T | G | C |
| A1 | A | G | G | G | CTTAG | G | A | C | T | G | T | T | C |
| A2 | A | G | G | G | CTTAG | G | A | C | T | G | T | T | C |
| A3 | A | G | G | G | CTTAG | G | A | C | T | G | T | T | C |
| A4 | A | **-** | G | G | CTTAG | G | A | C | T | G | T | T | C |
| A5 | A | **-** | G | G | CTTAG | G | A | C | T | G | T | T | C |
| A6 | A | **-** | G | G | CTTAG | G | A | C | T | G | T | T | C |
| A7 | A | G | G | G | CTTAG | G | A | C | T | G | T | T | C |
